# A technical review of multi-omics data integration methods: from classical statistical to deep generative approaches

**DOI:** 10.1093/bib/bbaf355

**Published:** 2025-08-01

**Authors:** Ana R Baião, Zhaoxiang Cai, Rebecca C Poulos, Phillip J Robinson, Roger R Reddel, Qing Zhong, Susana Vinga, Emanuel Gonçalves

**Affiliations:** INESC-ID, Rua Alves Redol 9, 1000-029 Lisboa, Portugal; Instituto Superior Técnico (IST), Universidade de Lisboa, Av. Rovisco Pais, 1049-001 Lisboa, Portugal; ProCan®, Children’s Medical Research Institute, Faculty of Medicine and Health, The University of Sydney, 214 Hawkesbury Road, Westmead, NSW 2145, Australia; ProCan®, Children’s Medical Research Institute, Faculty of Medicine and Health, The University of Sydney, 214 Hawkesbury Road, Westmead, NSW 2145, Australia; ProCan®, Children’s Medical Research Institute, Faculty of Medicine and Health, The University of Sydney, 214 Hawkesbury Road, Westmead, NSW 2145, Australia; ProCan®, Children’s Medical Research Institute, Faculty of Medicine and Health, The University of Sydney, 214 Hawkesbury Road, Westmead, NSW 2145, Australia; ProCan®, Children’s Medical Research Institute, Faculty of Medicine and Health, The University of Sydney, 214 Hawkesbury Road, Westmead, NSW 2145, Australia; INESC-ID, Rua Alves Redol 9, 1000-029 Lisboa, Portugal; Instituto Superior Técnico (IST), Universidade de Lisboa, Av. Rovisco Pais, 1049-001 Lisboa, Portugal; IDMEC, Instituto Superior Técnico, Universidade de Lisboa, Av. Rovisco Pais, 1049-001 Lisboa, Portugal; INESC-ID, Rua Alves Redol 9, 1000-029 Lisboa, Portugal; Instituto Superior Técnico (IST), Universidade de Lisboa, Av. Rovisco Pais, 1049-001 Lisboa, Portugal

**Keywords:** deep generative models, machine learning, multi-omics integration, precision medicine

## Abstract

The rapid advancement of high-throughput sequencing and other assay technologies has resulted in the generation of large and complex multi-omics datasets, offering unprecedented opportunities for advancing precision medicine. However, multi-omics data integration remains challenging due to the high-dimensionality, heterogeneity, and frequency of missing values across data types. Computational methods leveraging statistical and machine learning approaches have been developed to address these issues and uncover complex biological patterns, improving our understanding of disease mechanisms. Here, we comprehensively review state-of-the-art multi-omics integration methods with a focus on deep generative models, particularly variational autoencoders (VAEs) that have been widely used for data imputation, augmentation, and batch effect correction. We explore the technical aspects of VAE loss functions and regularisation techniques, including adversarial training, disentanglement, and contrastive learning. Moreover, we highlight recent advancements in foundation models and multimodal data integration, outlining future directions in precision medicine research.

## Introduction

**Figure 1:**
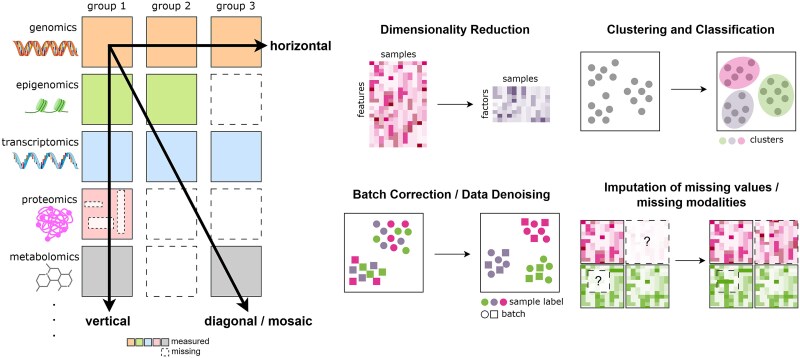
Multi-omics data integration. Left: Illustration of diverse omics layers (rows) for three different groups of samples (columns), highlighting four integration strategies. Vertical integration combines different omics modalities within the same group of samples; horizontal integration aligns datasets from the same omics layer across different sample groups (e.g. batches, cellular models), typically addressing batch effect correction; diagonal integration combines distinct omics modalities from different sample groups to explore inter-modality relationships across groups, and mosaic integration leverages overlapping modalities across samples to infer relationships and impute missing modalities. Dashed boxes indicate missing data. Right: Overview of common tasks in multi-omics analysis. Dimensionality reduction infers low-dimensional embeddings that facilitate downstream tasks like clustering and classification. Batch effect correction ensures that samples cluster based on biological attributes, such as tissue or cell type, rather than technical artifacts. Imputation addresses missing data, both for randomly missing features and for entire missing modalities. Symbol? Inside dashed boxes denote missing values or modalities.

Recent advances in high-throughput technologies have enabled the comprehensive characterisation of cellular models across multiple molecular layers—omics (Fig. 1). Multi-omics studies have become commonplace in precision medicine research, providing a holistic perspective of biological systems [1, 2], uncovering disease mechanisms, identifying molecular subtypes [3–5], and discovering new drug targets [6] and biomarkers for clinical applications [7–14].

Several consortia, including TCGA/ICGC [15] and ProCan [16], have generated invaluable multi-omics datasets and resources, particularly for cancer studies. Multi-omics data repositories and portals were reviewed in [17–19]. Despite the potential, integrating these datasets remains challenging due to their high-dimensionality, heterogeneity, and sparsity [20, 21]. Multi-omics datasets often comprise thousands of features and are generated through diverse laboratory techniques, leading to inconsistent data distributions [20, 22]. Moreover, due to experimental limitations, data quality issues, or incomplete sampling, these datasets are often unbalanced and incomplete [23].

To address these issues, statistical and machine learning models focusing on dimensionality reduction, batch effect correction, and data imputation have been developed (Fig. 1). Dimensionality reduction techniques infer low-dimensional spaces that capture variability across omics, facilitating downstream tasks like clustering and classification [24, 25]. Batch effect correction attenuates technical biases while preserving critical biological signals [26, 27] and imputation techniques enhance data quality through data denoising and augmentation [28–30].

Many authors have reviewed multi-omics integration methods, offering diverse perspectives on approaches, challenges, and applications. Several of these reviews have focused on state-of-the-art statistical and machine learning approaches, categorising multi-omics integration methods based on different criteria. For instance, some classify these methods by the intrinsic nature of multi-omics experiments [31, 32], while others focus on fusion strategies [21, 33]. Other authors emphasized the applications of multi-omics integration methods and the supported omics data types [17, 19], with a particular focus on oncology [11, 18, 34, 35]. In Vahabi and Michailidis [36], unsupervised learning methods were reviewed based on their underlying approach. Recently, deep learning-based approaches have gained prominence, and several authors reviewed both traditional architectures, emerging trends, and their applications [37–46].

**Figure 2:**
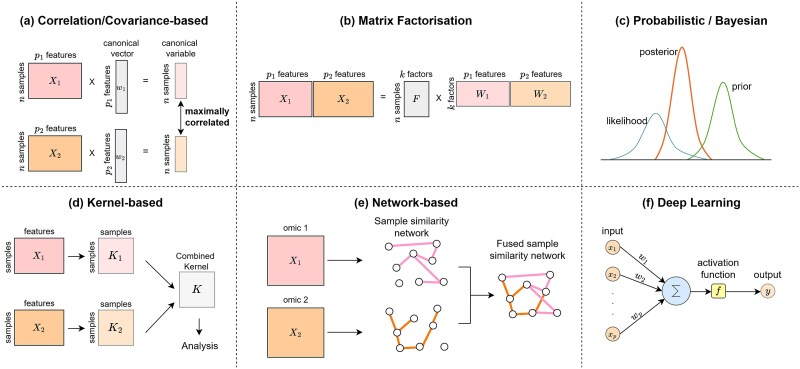
Schematic representation of the multi-omics integration approaches reviewed. (a) Canonical correlation analysis; (b) matrix factorisation of omics matrices into a shared and omics-specific matrices; (c) Bayesian approaches for probabilistic modelling; (d) multiple kernel learning; (e) similarity network fusion; (f) artificial neuron, the fundamental unit of neural networks: For each sample vector each input feature ${\mathrm{x}}_{\mathrm{i}}$ is associated with a weight ${\mathrm{w}}_{\mathrm{i}}$. To compute a weighted sum. The resulting value is passed through an activation function to produce the neuron’s output.

This review provides a comprehensive technical overview of the methods developed for multi-omics data integration, categorising them into correlation-based, matrix factorisation, probabilistic, network, kernel-based, or deep learning approaches (Fig. 2, Table 1). Instead of reviewing a specific model category, multi-omics data types, or biological applications, we emphasize the architectural and computational innovations driving this broad range of methods and categories. Recent advances in the field have shifted the focus from more classical statistical to deep learning approaches, particularly generative methods. Therefore, we place particular emphasis on variational autoencoders (VAEs), which have gained prominence since 2020 for tasks such as imputation, denoising, and creating joint embeddings of multi-omics data [47–51]. Beyond describing VAE applications, we explore training strategies and regularisation techniques proposed for adversarial training, cycle-consistency, contrastive, and disentangled representation learning.

**Table 1:** Overview of multi-omics integration methods categories, outlining their general strengths, limitations, and typical applications.

Model approach	Strengths	Limitations	Typical applications in multi-omics analysis
Correlation / Covariance-based	Captures relationships across omics based on correlation or covariance, interpretable, flexible sparse and regularised extensions	Limited to linear associations, typically requires matched samples across omics	Disease subtyping, detection of co-regulated modules
Matrix Factorisation	Efficient dimensionality reduction, identifies shared and omic-specific factors, interpretable, scalable	Assumes linearity, does not explicitly model uncertainty or noise	Disease subtyping, identification of shared molecular patterns, biomarker discovery
Probabilistic-based	Efficient dimensionality reduction, captures uncertainty in latent factors, probabilistic inference	Computationally intensive, may require careful tuning and strong model assumptions	Disease subtyping, latent factors discovery, biomarker discovery
Multiple Kernel Learning	Can capture nonlinear relationships, well-suited for heterogeneous data types	Sensitive to kernel choice and parameters, limited interpretability	Disease subtyping, patient similarity analysis
Network-based	Represents samples or omics relationships as networks, typically robust to missing data	Sensitive to similarity metrics choice, may require extensive tuning	Disease subtyping, patient similarity analysis, identification of regulatory mechanisms
Deep generative learning	Learns complex nonlinear patterns, flexible architecture designs, can support missing data and denoising	High computational demands, limited interpretability, requires large data to train	High-dimensional omics integration, data augmentation and imputation, disease subtyping, biomarker discovery

Here, we standardize terminology and provide clarity in a field with a wide range of different methods and definitions. Finally, we highlight promising future directions, including foundation models and multimodal data integration, which have the potential to advance the field and further enhance its impact on precision medicine.

## Classical statistical and machine-learning approaches

In this section, we introduce multi-omics integration methods, ranging from correlation and covariance-based to matrix decomposition methods, and probabilistic or Bayesian approaches. Lastly, network and kernel-based methods are highlighted. Generally, this considers $ M $ different omics matrices $ {X}_i\in{\mathbb{R}}^{n_i\times{p}_i} $, $ i=1,\dots, M $ each with $ {n}_i $ samples and $ {p}_i $ features.

### Correlation/covariance-based methods

Canonical Correlation Analysis (CCA) [52] is a classical statistical method designed to explore the relationships between two sets of variables ${X}_1$ and ${X}_2$ ($M=2$), with the same set of samples $n.$ CCA aims to find column vectors ${w}_1\in{\mathbb{R}}^{p_1}$and​ ${w}_2\in{\mathbb{R}}^{p_2}$that maximise the correlation between the linear combinations ${X}_1{w}_1$ and ${X}_2{w}_2$:


(1) \begin{equation*} \mathrm{argma}{\mathrm{x}}_{w_1,{w}_2}\ \mathrm{corr}\left({X}_1{w}_1,{X}_2{w}_2\right), \end{equation*}

where ${w}_1$and ${w}_2$ are the first canonical vectors, and ${X}_1{w}_1$ and ${X}_2{w}_2$ are the corresponding canonical variables (Fig. 2a) used for further multi-omics analysis.

CCA has proven particularly useful as a joint dimensionality reduction and information extraction method in genomic studies, where multiple types of data, such as DNA copy number or mutation, are often collected from the same set of samples [53–57]. However, the high-dimensional nature of multi-omics data presents a major challenge, often resulting in ill-defined problems that limit the direct applicability of classical CCA. Therefore, several CCA extensions were proposed to induce sparsity in the solution of the optimisation problem [54–56, 58, 59]. To extend the application of CCA-based methods to more than two datasets, sparse and regularized Generalised CCA (sGCCA/rGCCA) were proposed [60, 61], being currently one of the most widely used generalisations of CCA to multi-omics data.

DIABLO [62] extends sGCCA to a supervised framework. It simultaneously maximizes common or correlated information between multiple omics datasets and minimizes the prediction error of a response variable. This approach is particularly effective for selecting co-varying modules that explain the outcome, usually phenotypic traits.

Recently, deep learning-based extensions of traditional CCA have been proposed to handle nonlinearity and scalability in multi-omics data integration, including SDGCCA (Supervised Deep GCCA) [63], and VIPCCA [64] or VIMCCA [65] for unpaired and paired single-cell data, respectively.

Partial Least Squares (PLS) is an alternative approach for data integration that aims to maximize the covariance between components. Various methods were also proposed to find sparse solutions [66] and to extend the application to more than two datasets [67]. Several implementations of PLS, which optimize different objective functions with different constraints, have been described and reviewed in [68].

### Matrix factorisation methods

Matrix decomposition is a powerful method for joint dimensionality reduction, condensing datasets into fewer factors to reveal important patterns that can be used, e.g. to identify disease-associated biomarkers or cancer subtypes (Table 1).

JIVE [69] is considered an extension of Principal Component Analysis (PCA) that decomposes each omics matrix into joint and individual low-rank approximation and a residual noise by minimising the overall sum of squared residuals. JIVE quantifies the variation across and within datasets, reduces the dimensionality of the data, and avoids overfitting.

Non-Negative Matrix Factorisation (NMF) is a popular technique for decomposing datasets into two non-negative matrices. Several extensions of NMF have been developed to address the specific challenges of multi-omics datasets.

jNMF [70] decomposes multiple omics datasets into a shared basis matrix $F\in{\mathbb{R}}^{n\times k}$ and specific omics coefficient matrices ${W}_i\in{\mathbb{R}}^{k\times{p}_i}$ (Fig. 2b):


(2) \begin{equation*} {X}_i\approx F{W}_i. \end{equation*}

The objective function is formulated as ${\min}_{F,{W}_i}\ {\sum}_{i=1}^M{\left\Vert{X}_i-F{W}_i\right\Vert}_{\mathrm{F}}^2,\\ F,{W}_i\ge 0$, where ${\left\Vert .\right\Vert}_{\mathrm{F}}$​ denotes the Frobenius norm, and the constraint $F,{W}_i\ge 0$ ensures that all entries in matrices $F$ and ${W}_i$ are non-negative.

intNMF [71] is an extension of NMF for clustering analysis of multi-omics data. Once the matrix $F$ has been computed, each sample is associated with one of the $k$ clusters, determined by the highest entry in the matrix.

For single-cell data, LIGER [72] applies iNMF [73] to decompose each omics dataset into dataset-specific weights (${V}_i$), shared weights ($W$), and sample specific factors (${F}_i$). The objective function is defined as:


(3) \begin{equation*} {\min}_{F_i,W,{V}_i}\ {\sum}_{i=1}^M{\left\Vert{X}_i-{F}_i\left(W+{V}_i\right)\right\Vert}_{\mathrm{F}}^2+\lambda{\sum}_{i=1}^M{\left\Vert{F}_i{V}_i\right\Vert}_{\mathrm{F}}^2,{F}_i,W,{V}_i\ge 0. \end{equation*}

An additional regularisation term is added to handle omics-specific noise and heterogeneity. After performing iNMF, each cell is assigned to the factor with the highest loading, and a shared-factor neighborhood graph is constructed by connecting cells with similar factor loading profiles, improving the robustness of joint clustering. UINMF [74] extends iNMF by adding an unshared weights matrix term to the objective function. This method incorporates features that belong to only one or a subset of the omics datasets, performing mosaic integration.

### Probabilistic-based methods

Matrix factorisation is a robust approach for dimensionality reduction but has several limitations, particularly in handling missing data. Probabilistic matrix factorisation offers substantial advantages by incorporating uncertainty estimates and allowing for flexible regularisation (Table 1).

iCluster [75] is a joint latent variable model designed to identify latent cancer subtypes based on multi-omics data. This method decomposes each omics into a shared latent factor matrix $F\in{\mathbb{R}}^{n\times k}$ and omics-specific weight matrices ${W}_i\in{\mathbb{R}}^{k\times{p}_i}$:


(4) \begin{equation*} {X}_i=F{W}_i+{E}_i, \end{equation*}

assuming both the errors ${E}_i$ and the latent factor matrix $F$ follow a normal distribution. Latent variables are estimated using the expectation-maximisation method [76], and clusters are obtained by applying K-means to matrix $F$. iClusterPlus [77] extends iCluster by modelling different statistical distributions to handle discrete omics datasets but it was criticized for its computational intensity. iClusterBayes [78] further extends iClusterPlus by introducing a fully Bayesian inference approach, improving both stability and computational efficiency.

LRAcluster [79] is another example of a clustering probabilistic method for continuous and categorical data integration, but it is based on a low-rank probabilistic approach to improve latent variables estimation efficiency. moCluster [80] is also a joint latent model that uses modified consensus PCA [81] for latent variable estimation, providing a more stable and efficient alternative to the expectation-maximisation algorithm used in iCluster.

MOFA [82] is a probabilistic Bayesian framework for multi-omics integration, designed to handle diverse data distributions and missing values automatically (Fig. 2b-c). It decomposes omics datasets as in Equation 4, placing prior distributions on all unobserved variables. To enhance interpretability and disentangle variation across omics, MOFA employs a two-step regularisation process to enforce sparsity in the weight matrix and capture both omics-specific and shared factors. MOFA is optimized by maximising the Evidence Lower Bound (ELBO) [83], enhancing its generative capabilities. MOFA+ [84] further extends this framework to improve scalability for both bulk and single-cell datasets.

### Kernel-based methods

The previously described models rely predominantly on linear combinations to integrate multi-omics data. In contrast, kernel- and network-based approaches enable the modelling of nonlinear relationships across omics layers in a structured way.

Kernel learning approaches [85, 86] use kernel functions to map omics data into higher-dimensional feature spaces. This mapping is defined by a kernel matrix that represents pairwise similarities, computed as their inner product in the feature space ${K}_{i,j}=k\left({x}_i,{x}_j\right)=\left\langle \phi \left({x}_i\right),\phi \left({x}_j\right)\right\rangle$, where $\phi$ maps the original data to the feature space. The kernel function $k\left({x}_i,{x}_j\right)$ is the only required definition for the kernel method. In multi-omics integration, a kernel matrix is computed for each omics layer and multiple kernel learning (MKL) combines them into a final kernel matrix by minimising an objective function. The final kernel matrix is used for downstream analysis (Fig. 2d). In MKL, the integration problem shifts from heterogeneous feature spaces to a unified sample space.

Several MKL-based methods have been proposed for multi-omics integration. For instance, rMKL-LPP [87] uses a linear combination of kernels constructed using an objective function based on the Locality Preserving criterion, while web-rMKL [88] provides a web-based implementation. pairwiseMKL [89] is a time and memory-efficient version of MKL with applications to drug response prediction.

### Network-based methods

Network-based methods leverage graphs and other network structures to represent omics data and their relationship, capturing topological structures and interactions to identify key biomarkers and reveal biological pathways. Similarity Network Fusion (SNF) [90] is a popular network-based method for multi-omics integration. SNF constructs patient similarity networks for each omics layer, where nodes represent samples and edges their similarity. These networks are iteratively fused into a unified similarity network using a nonlinear combination method based on message passing theory (Fig. 2e). The unified similarity network captures shared patterns across omics layers, enhancing subtype identification for several diseases [91–94]. As MKL, SNF is computationally efficient as it depends primarily on the number of samples rather than features. However, it does not distinguish between data types and relies on Euclidean distance to calculate sample similarity, which may not fully capture the complex relationships in omics data. Several extensions and deep learning-based adaptations have been proposed to overcome these limitations [95–99].

NEMO [100] is a popular similarity network-based method that handles unmatched samples without requiring imputation. It constructs a patient similarity matrix for each omics using a radial basis function kernel, adjusts for local neighborhoods, and computes an average relative similarity matrix shared across all omics. Spectral clustering is then applied to identify disease subtypes, demonstrating superior clustering performance compared to other state-of-the-art methods.

Overall, similarity kernel and network-based methods focusing on sample similarities and relationships offer a flexible strategy to integrate heterogeneous datasets (Table 1).

## Deep learning approaches

Deep learning approaches have emerged as powerful and flexible tools for multi-omics integration, capturing nonlinear and complex patterns from data (Table 1). The following sections explore various neural network architectures for multi-omics integration, from non-generative to generative approaches, placing particular focus on VAEs.

### Non-generative models

Non-generative approaches focus on learning direct mappings or relationships between input features and outputs, often prioritising tasks like classification, regression, or dimensionality reduction.

#### Feed forward neural networks

The feed-forward neural network (FFNN) is the most common neural network architecture, consisting of fully interconnected layers of neurons. The individual neurons compute weighted sums of their inputs, apply an activation function and propagate it forward to the next layer (Fig. 2f). The activation function typically introduces nonlinearity, increasing the expressive power of the network. These models are trained to minimize a loss function using optimisation techniques such as backpropagation. Despite being computationally intensive and having a high number of parameters, neural networks have demonstrated significant potential in biological data analysis [101, 102].

MOLI [103] is a supervised FFNN designed for drug response prediction. This method employs separate subnetworks for each omics layer, extracts features, and concatenates them into a unified representation for the final classification network. MOLI incorporates a binary cross-entropy and a triplet loss function for training. FFNNs have also been applied to other tasks, such as synergistic drug combination prediction [104, 105], survival analysis [106] or trajectory inference [107].

#### Graph neural networks

Graph neural networks (GNNs) are a powerful framework for processing data structured as graphs, making them particularly valuable in biological research where entities are intrinsically linked, such as in protein–protein interactions (PPIs) or gene regulatory networks. Graph Convolutional Networks (GCNs) are the most dominant GNNs introducing convolution operations to the graph structure. MOGONET [108] is designed for supervised multi-omics integration and classification by constructing a sample similarity network for each omics and leveraging GCNs to predict labels based on individual modalities. scMoGNN [109] is an example of a framework that leverages GNNs for single-cell multi-omics data integration to tackle modality prediction, matching, and joint embedding tasks.

While methods like MOGONET focus on sample similarity networks, they do not incorporate biological interaction data, such as PPIs, which could provide additional meaningful context. For instance, Zhuang et al. [110] proposed a GCN method for disease classification integrating transcriptomics and proteomics data with PPI networks.

#### Autoencoders

Autoencoders are an unsupervised deep learning model widely used for dimensionality reduction and feature extraction tasks—embeddings. This model architecture leverages neural networks to compress the input data into a lower dimensional latent space via an encoder and attempts to reconstruct it back to the original space through a decoder. Several autoencoder-based models have been developed for multi-omics integration for cross-modality translation tasks [111, 112] or for joint dimensionality reduction, where the latent space is used for downstream tasks like disease prognosis and subtyping [113–118], clustering [119, 120], synergistic drug combination prediction [121], or batch correction [122, 123].

Among several extensions of autoencoders, VAEs [124, 125] are the most prominent for multi-omics data analysis due to their probabilistic framework and generative capability.

#### Variational autoencoders

In contrast with non-generative approaches, deep generative models (DGMs) learn the underlying data distribution, enabling the generation of realistic synthetic data. DGMs have significantly impacted molecular biology, including the multi-omics data integration field [126]. The majority of DGMs published and reviewed here are based on VAEs [124, 125]. However, other frameworks including generative adversarial networks (GANs) [127] and, more recently, generative pre-trained transformer (GPT) approaches [128] have been proposed.

In a common Bayesian approach, each sample vector $x\in{\mathbb{R}}^p$, with $p$ features, is assumed to be generated by a latent vector $z\in{\mathbb{R}}^k$, with $k\ll p$. The latent vector is drawn from a prior distribution ${p}_{\theta }(z)$, and the sample vector is generated from the conditional distribution ${p}_{\theta}\left(x|z\right)$, where θ are the parameters of the decoder network. Since the estimation of the marginal data likelihood ${p}_{\theta }(x)$ is computationally intractable, approximation inference methods are employed to efficiently estimate model parameters.

**Figure 3:**
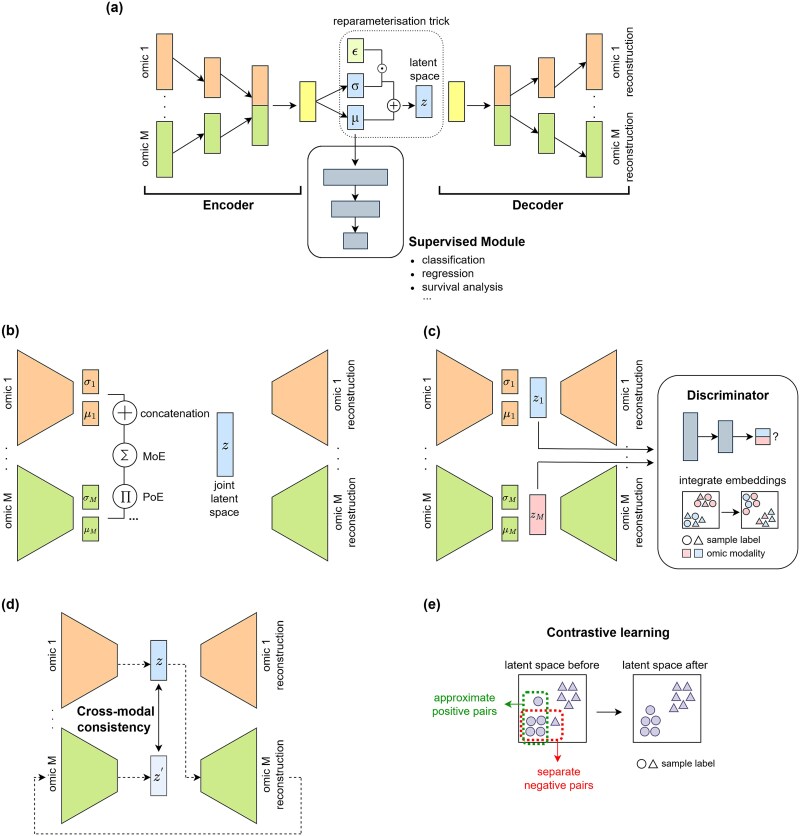
VAE architectures. (a) VAE with a supervised module for task-specific supervision. Each rectangle represents a fully connected block. Data from two omics are concatenated in the second hidden layer. The parameters $\mathrm{\mu}$ and $\mathrm{\sigma}$ represent the mean and standard deviation of the learned posterior distribution, and $\mathrm{\varepsilon} \sim \mathcal{N}\left(0,1\right)$. The reparameterisation trick is depicted in the dashed box. (b) Multimodal VAE architecture, highlighting three strategies to build the joint latent space: concatenation, MoE, and PoE. (c) Adversarial training strategies in VAEs to align the latent spaces of different omics modalities. (d) Cross-modal cycle consistency. (e) Contrastive learning to self-supervise VAEs by gathering positive pairs and separating negative pairs.

VAEs provide a principled way for performing variational inference by approximating the true posterior distribution ${p}_{\theta}\left(z|x\right)$ using a variational posterior ${q}_{\phi}\left(z|x\right)$, where $\phi$ are the parameters of the encoder. This encoder network processes the input data and outputs two layers representing the mean μ and standard deviation σ of the variational posterior. The reparameterisation trick is used to allow backpropagation by sampling $z$ as $z=\mu +\sigma \varepsilon$, $\mathcal{\varepsilon}\sim \mathcal{N}\left(0,1\right)$. Finally, the latent vector is the input of the decoder network that will reconstruct the input data (Fig. 3a).

The parameters $\phi$ and $\theta$ of the encoder and decoder networks, respectively, are jointly optimised by maximising the ELBO:


(5) \begin{equation*} ELB{O}_{VAE}\left(x,\phi, \theta \right)={\mathbb{E}_{q_{\phi}\left(z|x\right)}\left[\log{p}_{\theta}\left(x|z\right)\right]-\lambda\{D}_{KL}\big({q}_{\phi}\left(z|x\right)\Vert{p}_{\theta }(z)\big), \end{equation*}

where ${D}_{KL}$ is the Kullback–Leibler (KL) divergence, weighted by the hyperparameter $\lambda$. The overall VAE loss function is the negative ELBO. The first term aims to minimise the decoder reconstruction error, e.g. using the mean squared error. The KL divergence term regularizes the model by minimising the divergence between the variational posterior and prior distribution. The variational posterior typically follows a Gaussian distribution. However, in single-cell data applications other probability distributions, such as the negative binomial, can better handle the sparsity and count nature of the data [129, 130].

VAEs are widely used for multi-omics analysis, particularly for single-cell experiments, due to their flexibility in handling high-dimensional and incomplete datasets while balancing dimensionality reduction and generative capabilities. The following sections detail various methodological improvements that have been proposed to enhance VAE integration performance during recent years (Table 2).

**Table 2:** Overview of deep generative models for multi-omics data integration. Omics modalities listed correspond to those demonstrated in the original publications, although some models may be extended to other omics layers. Similarly, the integration type refers to the categorisation provided by the authors or to the integration setting inferred from the original work; however, several methods may be adapted for alternative integration scenarios. CyTOF: cytometry by time of flight; CNV: copy number variation; scATAC-seq: single-cell sequencing assay for transposase-accessible chromatin; scRNA-seq: single-cell RNA-sequencing; snRNA-seq: single-nucleus RNA-sequencing; snmC-seq: single-nucleus methylation sequencing.

Name	Method	Omics modalities demonstrated	Integration type demonstrated	Key tasks and applications
*General multi-omics methods*				
OmiVAE / XOmiVAE [136,137]	VAE with a supervised module / extension incorporating Deep SHAP	gene expression, DNA methylation		joint embedding, classification, clustering
MMD-VAE [48]	VAE with a supervised module and a MMD instead of KL regularizer	DNA methylation, CNV, mRNA, or RNAseq		joint embedding, classification, survival analysis, clustering
OmiEmbed [139]	VAE with supervised modules	miRNA, gene expression, DNA methylation		joint embedding, classification, regression, survival prediction, clustering
omicsGAN [148]	GAN to integrate two omics modalities and their interaction networks	mRNA, miRNA expression		classification, survival prediction
MOSA [23]	Conditional MVAE with concatenation of latent spaces and a contrastive loss; SHAP values	CNV, proteomics, gene expression, methylomics, metabolomics, drug response, CRISPR-Cas9		joint embedding, data augmentation, clustering, biomarker identification related to drug and gene dependencies
*Single-cell multi-omics methods*				
scMVAE [142]	MVAE with three strategies of joint-learning (direct concatenation, neural network, PoE)	scRNA-seq, scATAC-seq	Vertical	joint embedding, data denoising and imputation, regulatory mechanisms analysis, clustering
scMM [129]	MVAE with MoE and a pseudo cell generation strategy for model interpretability	scRNA-seq, scATAC-seq, or surface protein		joint embedding, cross-modal generation, regulatory inference, clustering
DCCA [147]	Separate VAEs mutually supervised by cross-omics cycle attention	scRNA-seq, scATAC-seq		separate embeddings, transfer learning, data denoising, regulatory inference, missing cells and omics generation, clustering
scMVP [154]	MVAE with GMM prior, attention-based channels, and intra-modal consistency modules	scRNA-seq, scATAC-seq		joint embedding, clustering, trajectory inference, cis-regulatory element prediction, data denoising and imputation
scDisInFact [135]	VAE with an additional MMD term for disentanglement learning	scRNA-seq	Horizontal	shared-bio embedding, clustering, batch effect correction, condition-associated key gene detection, perturbation prediction
SCIM [47]	VAE with adversarial training to distinguish between omics based on latent space	scRNA-seq, CyTOF		joint embedding, cell matching across different omics technologies, batch correction, clustering
Cobolt [146]	MVAE with PoE for the integration of data from multi and single-modality platforms	scRNA-seq, scATAC-seq		joint embedding, clustering
Multigrate [49]	MVAE with a shared decoder, PoE strategy, and an additional MMD regularizer	scRNA-seq, scATAC-seq, or surface protein		joint embedding, multimodal reference atlas building, transfer learning for multi-omic query datasets mapping, imputation of missing omics, clustering
GLUE [149]	Omics-specific VAEs and a graph VAE; adversarial training to distinguish between omics based on cell embeddings	scRNA-seq, scATAC-seq, snmC-seq		joint embedding, regulatory inference, multi-omics atlas building, clustering
Portal [152]	Encoder and GAN: modality-specific encoders, cross-modal generators, and discriminators; additional regularizers for cross-modal embeddings and samples consistency, and intra-modality reconstructions	scRNA-seq, scATAC-seq, or snRNA-seq		joint embedding, batch correction, label transfer, clustering
sciCAN [155]	Encoder and GAN: one discriminator to distinguish between omics latent spaces, one discriminator to distinguish between real and cross-modal generated data, cross-modal embeddings cycle-consistency	scRNA-seq, scATAC-seq	Diagonal or Mosaic	joint embedding, co-trajectory inference, label transfer, clustering
scVAEIT [164]	VAE with conditional variational inference using missing masks	scRNA-seq, scATAC-seq, surface proteins		joint embedding, data denoising, imputation of missing features and omics modalities, transfer learning to new datasets, clustering
JAMIE [50]	VAEs with cross-modal correspondence and correlation-based latent aggregation; SHAP values	scRNA-seq, scATAC-seq, electrophysiology data		separate aggregate embeddings, cross-modal imputation, phenotype prediction, clustering
MultiVI [134]	MVAE using distributional average and penalisation to mix latent embeddings; symmetric Jeffrey’s divergence term; adversarial training to distinguish between batches or modalities based on the shared latent space	scRNA-seq, scATAC-seq, surface protein		joint embedding, differential expression analysis, imputation of missing features and omics modalities, batch correction, clustering
MIDAS [51]	MVAE with PoE that employs self-supervised modality alignment, information-theoretic latent disentanglement, and masking techniques to handle missing modalities	scRNA-seq, scATAC-seq, surface protein		joint embedding, batch correction, transfer learning, imputation of missing features and omics modalities, clustering, trajectory inference
scCross [150]	Omics-specific VAEs with an FFNN aligner; one discriminator to distinguish between omics based on the shared latent space and omics specific discriminators to distinguish between original and reconstructed data	scRNA-seq, scATAC-seq, snmC-seq		joint embedding, cross-modal generation, data simulation, in silico cellular perturbations, clustering

#### Maximum mean discrepancy regularizer

VAEs can face issues with the usual ELBO-based loss function, leading to failures in learning a variational posterior distribution that approximates the true posterior and meaningful or informative latent features [48, 131]. Inference failures can occur due to an imbalance in ELBO optimisation or modelling bias. When the input data is high-dimensional compared to the latent space, the model can prioritize data reconstruction over learning a distribution that approximates the true posterior, potentially leading to poor generalisation and overfitting. Additionally, VAEs can reconstruct input data without relying on the latent variables, making them uninformative and failing to capture meaningful information about the input data.

To address these issues, several models [48, 132] have replaced the traditional KL divergence term with the Maximum Mean Discrepancy (MMD) [133] in their loss function. The MMD-based regularizer estimates the divergence by how different the moments of two distributions $p(z)$ and $q(z)$ are and can be defined as:


(6) \begin{align*} \mathrm{MMD}\big(p(z)\Vert q(z)\big)=&\ {\mathbb{E}}_{p(z),p\left({z}^{\prime}\right)}\left[k\left(z,{z}^{\prime}\right)\right]+{\mathbb{E}}_{q(z),q\left({z}^{\prime}\right)}\left[k\left(z,{z}^{\prime}\right)\right] \nonumber \\ & -2\ {\mathbb{E}}_{p(z),q\left({z}^{\prime}\right)}\left[k\left(z,{z}^{\prime}\right)\right], \end{align*}

where $k\left(z,{z}^{\prime}\right)$ is any positive-definite kernel, with the Gaussian kernel a popular choice.

Several models reviewed in the following sections incorporate MMD terms into their loss functions. For instance, MMD-VAE [48] replaces the KL divergence with an MMD regularizer. Multigrate [49] and MultiVI [134] add MMD terms into the VAE loss to ensure alignment and consistency across omics modalities. Additionally, scDisInFact [135] introduces a MMD term to ensure disentanglement of latent factors.

#### Supervised learning tasks with VAEs

In a standard VAE, the bottleneck layer extracts essential features for accurate input data reconstruction. However, these features are often too general and may not be relevant to particular downstream analyses. To address this, several VAE-based models incorporate supervised modules. These models typically connect the mean vector $\mu$ or the latent space $z$ to a neural network to perform a specific task (Fig. 3a). This introduces a task-specific regularizer, by adding the loss of the downstream task into the overall VAE loss function. The total loss ensures the latent factors extracted by the VAE are informative for both accurate data reconstruction and supervised tasks.

One example is OmiVAE [136], which integrates gene expression and DNA methylation data to classify pan-cancer tumour samples. XOmiVAE [137] extends this by incorporating explainability through deep SHAP [138], providing insights into individual features and latent dimensions contributions for cancer classification to identify potential biomarkers.

OmiEmbed [139] extends previous models by incorporating additional downstream tasks such as demographic and clinical features reconstruction and survival prediction using a multi-task training strategy. The latent space is shared across tasks and the downstream module loss is the weighted sum of all downstream losses, improving the overall model performance through joint learning.

MMD-VAE [48] integrates tri-omics data for ovarian cancer analysis, focusing on molecular subtypes clustering, classification, and survival prediction. By replacing the KL divergence with an MMD regularizer, MMD-VAE improves performance on most omics datasets.

Overall, VAEs with supervised modules improve omics analysis by integrating generative modelling with supervised learning. This approach enhances the extraction of biologically relevant features and optimizes classification or regression performance, addressing limitations of traditional unsupervised methods.

#### Inferring joint latent representations with multimodal VAEs

Multimodal VAEs (MVAEs) are a common approach for multi-omics integration where each omics modality is assigned its own encoder-decoder and a shared latent space is inferred. There are several strategies to combine the latent variables from individual modalities into a unified latent representation, including concatenation or probabilistic methods such as the mixture of experts (MoE), product of experts (PoE) [140], and mixture-of-product-of-experts [141] (Fig. 3b).

In MoE approaches, the joint variational posteriors for $M$ omics modalities is defined as ${q}_{\phi}\left(z|{x}_{1:M}\right)={\sum}_{i=1}^M{\alpha}_i{q}_{\phi_i}\left(z|{x}_i\right)$, usually with ${\alpha}_i=\frac{1}{M}$, where ${x}_i$ represents a sample vector for omics $i$. The resulting ELBO is the weighted average of each modality’s ELBO. The PoE is an alternative approach that infers the joint posterior as the product of the individual variational posteriors ${q}_{\phi}\left(z|{x}_{1:M}\right)={\prod}_{i=1}^M{q}_{\phi_i}\left(z|{x}_i\right)$.

scMVAE [142] integrates multi-omics data with a multimodal encoder that infers the joint latent space with three different strategies: direct concatenation, neural network-based feature concatenation, and PoE. scMVAE includes single-modal encoders/decoders for data normalisation, denoising, and imputation and a Gaussian Mixture Model (GMM) as the prior to learn more disentangled and interpretable latent representations.

scMM [129] extends scMVAE using modality-specific encoders and decoders and a MoE strategy to infer the joint latent space. scMM enhances interpretability by generating pseudo cells with controlled variations in one latent dimension while keeping others fixed (latent traversals) and assessing correlations between traversed dimensions and features in each omics, identifying features strongly associated with each latent dimension. Additionally, scMM enables bidirectional cross-modal generation, allowing missing data in one modality to be inferred from another.

Multigrate [49] also applies a PoE approach for mosaic integration but is trained conditionally on a set of study labels. One key innovation is an additional MMD loss to minimize the distance between joint latent representations for pairs of datasets. The model architecture features modality-specific encoders and decoders, along with a shared decoder that captures both common biological and omics-specific patterns. Multigrate allows multimodal reference building, query data mapping into a reference atlas using transfer learning [143], and missing modalities imputation.

MultiVI [134] from the scvi-tools library [144] is built on earlier VAE-based models [130, 145] and is conceptually similar to Cobolt [146], a MVAE that employs a PoE approach. However, MultiVI is trained conditionally on a set of covariates, uses tailored noise models for each omics and infers the latent representation using a distributional average and penalisation strategy. For two omics modalities sample vectors ${x}_1$ and ${x}_2$, the shared latent space is defined as:


(7) \begin{equation*} z={w}_1{z}_1+{w}_2{z}_2\kern1em \mathrm{with}\kern1em {w}_1+{w}_2=1, \end{equation*}

where ${w}_1$ and ${w}_2$ are the weights for each modality, sample-specific or the same for all samples, also optimised during training. If only one modality is available for a specific sample, the latent space is inferred directly from that modality. MultiVI also minimises the distance between two latent representations using symmetric Jeffrey’s divergence:


(8) \begin{equation*} {L}_{symmKL}={D}_{KL}\big(q\left({z}_1\right)\Vert q\left({z}_2\big)\right)+{D}_{KL}\big(q\left({z}_2\right)\Vert q\left({z}_1\right)\big) \end{equation*}

An alternative MMD penalty was also explored by the authors. Additionally, MultiVI incorporates an adversarial penalty in the loss function, as detailed in the following sections, to minimize batch effects.

The projection of multi-omics data into a common latent space using MVAEs has become the dominant strategy for integration. The unified latent representation captures shared biological patterns across omics and facilitates downstream analyses such as clustering and visualisation. However, joint embeddings inevitably attenuate omic-specific patterns, potentially hiding relevant molecular insights. Therefore, several models have incorporated cross-learning approaches to retain omics-specific patterns while leveraging shared information.

#### Cross-learning approaches

The authors of scMVAE have extended this model to address limitations such as the need for all modalities to be present during training and omics-specific patterns attenuation. Therefore, DCCA [147] was proposed to combine multiple modalities into separate but coordinated latent space. DCCA processes each omics modality with a separate VAE that learns from each other with mutual supervision through cross-omics attention transfer. In DCCA, a well-trained teacher network on one modality guides the training of a student network of another modality. The model employs an additional term to each VAE loss to minimize the differences between the latent variables of each VAE, ensuring embeddings alignment:


(9) \begin{equation*} {L}_{DCCA- alignment}=\beta\ {\sum}_{i=1}^k{\left\Vert {z_2}^i-{z_1}^i\right\Vert}_2, \end{equation*}

where ${z}_1$ and ${z}_2$ are $k$-dimensional latent vectors for two omics modalities, and $\beta$ the weight of the added term.

JAMIE [50] is a VAE framework for di-omics integration and imputation. It uses a cross-modal correspondence matrix $F$ to handle partially aligned samples. JAMIE processes each modality with separate encoders, and their latent spaces are combined using correlation-based latent aggregation based on matrix $F$. Combination and alignment terms are added to the common VAE loss. The combination loss enforces similarity between separate and aggregate latent spaces, while the alignment term shapes the aggregated latent spaces to ensure similarity between cross-modal cell representations. JAMIE supports imputation and the prioritisation of input features for cross-modal imputation using SHAP. Its ability to adaptively learn correspondences and generate aggregate latent spaces makes it a versatile tool for multi-omics analysis.

#### Adversarial training strategies

With the advance of DGMs, GANs have inspired multi-omics integration methods by leveraging adversarial training to generate realistic synthetic data while distinguishing it from real data. GAN [127] architecture consists of two competing neural networks jointly optimized: a generator that learns to transform input noise into real data, and a discriminator that distinguishes between real and synthetic generated data.

omicsGAN [148] leverages Wasserstein GANs to integrate two omics modalities and their interaction networks to learn inter-modality relationships. The generator synthesizes data for one modality using the other modality and the interaction network adjacency matrix and the discriminator is adversarially trained to distinguish real from synthetic data.

Despite their flexibility and ability to learn complex data distributions, GANs face challenges such as the complexity of training dual networks, scalability issues with larger numbers of modalities, and the need for large sample sizes. To address these, adversarial training is often integrated into VAE frameworks as a regularisation strategy.

In this adversarial approach, a discriminator commonly distinguishes between omics modalities or batches based on latent space samples (Fig. 3c), while the encoder-decoders are trained to fool the discriminator by producing indistinguishable samples. The adversarial penalty in the standard VAE loss function encourages latent space alignment across modalities, attenuating batch effects.

SCIM [47] applies adversarial training for diagonal integration, aligning single-cell data from different omics technologies. Separate encoders generate a technology-invariant latent space using a discriminator to ensure that latent representations from different omics sources are comparable. Additionally, SCIM pairs cells across different technologies via their latent representations and a fast bipartite matching algorithm.

GLUE [149] also focuses on multi-omics diagonal integration using graph-guided embeddings and adversarial alignment. It employs omics-specific VAEs to learn sample latent spaces and a graph VAE incorporating prior biological knowledge to learn feature latent spaces for each omic. These latent spaces are combined via inner product to reconstruct omics data. A discriminator is used in the sample space to ensure cell embeddings alignment across omics and batch effects attenuation.

scCross [150] is a recent model designed for cross-modality translation and to perform *in silico* perturbations. scCross trains modality-specific VAEs to extract low-dimensional cell embeddings which are converted to a shared latent space using a FFNN aligner. A global discriminator operates on this shared latent space to identify the omics origin of the cells, ensuring proper alignment. For cross-modality translation, an omics-specific encoder maps input data into the shared latent space, which is then decoded into another omics modality. Modality-specific discriminators distinguish original from cross-modal reconstructed data. Several models have been developed for cross-modality translation [129, 151, 152], with scCross being one of the most recent.

#### Cycle-consistency training

Building on adversarial training in the latent space, cycle-consistency terms can also be incorporated into the VAE loss function to enforce intra-modal and cross-modal consistency, inspired by cycleGAN [153]. For intra-modal consistency, decoded representations are re-encoded with the omics-specific encoder and aligned with the original latent embeddings. For cross-modal consistency, embeddings from one modality are decoded and re-encoded using another modality’s encoder-decoder pair (Fig. 3d), ensuring that cross-modal translations remain consistent with the original latent space.

scMVP [154] is a VAE for di-omics vertical integration. It employs modality-specific encoders and decoders and a GMM prior to derive the shared latent space. This model uses multi-head self-attention transformer modules for scATAC-seq data to highlight the most informative features. Simpler attention blocks are used for scRNA-seq data to dynamically weight features, emphasising their importance during training. scMVP integrates single-modal encoders to ensure clustering consistency by minimising the KL-divergence between the joint embeddings and the modality-specific re-embeddings from the decoder output:


(10) \begin{equation*} {L}_{scMVP- consistency}\!=\!{D}_{KL}\big(q\left(z|{x}_1,{x}_2\right)\Vert q\left(\mathrm{z}|\hat{x_1}\right)\big)+{D}_{KL}\big(q\left(z|{x}_1,{x}_2\right)\Vert q\left(\mathrm{z}|\hat{x_2}\right)\big)\!\!, \end{equation*}

where ${x}_1,{x}_2$ are two different omics sample vectors, $\hat{x_1}$ and $\hat{x_2}$ the corresponding reconstructed vectors, and $z$ the common embedding. This intra-modal consistency loss ensures robust integration, the alignment of omics reconstructions and is used to impute missing data.

sciCAN [155] integrates two omics modalities by combining a shared encoder to derive latent representations with a cycle-GAN for modality alignment. The encoder uses noise contrastive estimation to preserve data structure and two discriminators are employed: one to distinguish between latent spaces of the two modalities and another to distinguish between real and generated data from a cross-modal generator. A cycle-consistency loss further ensures alignment between the embeddings produced by the generated data and the original ones.

Portal [152] performs di-omics integration using dual encoders, generators and discriminators. Encoders learn latent embeddings for each omics modality and the cross-modal generators generate synthetic omics data. Discriminators distinguish between original and generated data. To enhance consistency, Portal employs an autoencoder loss for intra-modality reconstruction, an alignment loss for consistency between the cross-modal embedding and the original one, and a cosine similarity loss to preserve correspondence between original and cross-modal reconstructed samples.

Adversarial and cycle consistency training strategies have proven effective in aligning and integrating multi-omics data by attenuating batch effects, encouraging consistency across modalities, improving reconstruction accuracy, and enabling the translation between different omics modalities.

#### Contrastive learning

Contrastive learning offers an alternative and complementary approach to extract meaningful representations in unsupervised learning methods. The main idea is to ensure that similar samples (positive pairs) are represented closer together in the latent space, while dissimilar samples (negative pairs) are pushed further apart [156, 157]. By enforcing these relationships during training, the model effectively learns to align similar sample types, improving accuracy and robustness in downstream tasks (Fig. 3e). To distinguish between positive and negative pairs, several metrics and functions can be used [157–159].

MOSA [23] is a conditional MVAE that adopts an embedding concatenation strategy to build the joint latent space. This model added a self-supervised contrastive loss to the standard VAE loss, defined as:


(11) \begin{equation*} {L}_{cosine- contrastive}={\left[{m}_{pos}-{s}_p\right]}_{+}+{\left[{s}_n-{m}_{neg}\right]}_{+}, \end{equation*}

where ${s}_p$ and ${s}_n$ represent the cosine similarity between positive pairs and negative pairs defined by whether two samples have the same tissue type, and ${m}_{pos}$ and ${m}_{neg}$ are positive and negative margins tuned during model training. Given that MOSA is one of the first models trying to systematically integrate over seven different omics, it adopts a whole-omic dropout layer strategy that masks entire omic during training, improving model generalization and reducing complexity. The model further enhances biological relevance by concatenating key features such as cancer driver mutations as conditionals to the model, and by incorporating SHAP for model interpretation.

Contrastive learning refines the representation of shared structures across omics modalities. However, to further unravel independent and interpretable factors driving biological variation, disentanglement representation learning (DRL) emerges as a complementary approach.

#### Disentanglement learning

DRL [160] is a machine learning strategy designed to extract latent representations that separate independent and informative factors of variation. VAEs are especially well-suited for DRL due to their flexibility, allowing the incorporation of regularizers to promote disentanglement [160, 161]. In multi-omics integration, DRL is particularly effective at disentangling complex molecular processes, improving interpretability and model generalisability.

scDisInFact [135] is a VAE framework designed to learn latent factors that disentangle conditions from batch effects in scRNA-seq data, enabling it to simultaneously remove batch effects and identify condition-associated key genes. The goal is to disentangle shared-bio factors (${z}_s$) and unshared-bio factors (${z}_u$) using two additional MMD loss terms. One term ensures that ${z}_s$ is independent of condition and batch labels:


(12) \begin{equation*} {L}_{\mathrm{MMD}- disentanglement}\left({z}_s\right)={\sum}_{c=1}^C\sum_{i\in{B}_c}\mathrm{MMD}\left({z}_s^{ref}\Big\Vert{z}_s^i\right), \end{equation*}

where ${B}_c$ is the set of batches under condition label $c$, and $C$ the total number of conditions. ${z}_s^{ref}$ is the latent representation of a reference batch and condition and ${z}_s^i$ the remaining ones. Similarly, the MMD term applied to ${z}_u$​ enforces independence from batch effects while preserving condition-specific variability:


(13) \begin{equation*} {L}_{\mathrm{MMD}- disentanglement}\left({z}_u\right)=\sum_{c=1}^C\sum_{i\in{B}_c}\mathrm{MMD}\left({z}_u^{ref(c)}\Big\Vert{z}_u^i\right), \end{equation*}

where ${z}_u^{ref(c)}$​ is the latent representation of a reference batch under condition label $c$. The disentanglement is further enhanced with group lasso for feature selection and cross-entropy loss for condition prediction using ${z}_u$.

MIDAS [51] is a MVAE employing the PoE approach for mosaic integration of single-cell data. It incorporates self-supervised learning and information-theoretic latent disentanglement [162] to perform dimensionality reduction, imputation, and batch correction. A disentanglement and an alignment term are added to the VAE loss. The disentanglement term is based on the Information Bottleneck loss and aims to disentangle the joint embedding into biological states and technical noise. MIDAS also highlights the need for approaches that can effectively handle missing modalities in mosaic multi-omics data.

#### Missing modalities in mosaic multi-omics data

Mosaic integration methods address the challenges of combining multi-omics datasets with incomplete and overlapping modalities across samples (Fig. 1), addressing scalability, cost, and modality coverage limitations. However, these methods face difficulties such as batch technical variation or robust imputation. Recent approaches, including matrix factorisation [74, 163], VAEs [49–51, 164], or StabMap [165], offer promising solutions.

For example, Multigrate [49] previously described takes the PoE approach and deals with the missing data by setting the posterior of the missing modality to 1. This allows the joint latent distribution to be determined from the available modalities, enabling joint embedding generation and reconstruction of all modalities, even if some are missing.

scVAEIT [164] uses conditional variational inference to handle missing features in multi-omics data. It generates random masks during training to encourage the model to predict the missing features based on the remaining observed data. This process allows the imputation of the unobserved values and denoising of the observed features.

MIDAS [51] addresses missing features by padding them with zeros for each cell, ensuring consistent feature vector size. The learned joint disentangled latent variables are passed to modality decoders, and a masking function removes the padded values, returning imputed values for missing features.

The flexibility of VAEs underscores their key role in advancing deep learning-based multi-omics integration, addressing challenges like handling missing modalities and enabling interpretable latent spaces. While these models provide robust frameworks for extracting meaningful biological insights, further refinement of their architectures is essential to enhance scalability, broaden applicability, and maximize their potential impact on precision medicine and synthetic biology.

## Discussion and promising perspectives

Multi-omics data integration is a rapidly advancing area in computational biology, driven by the need to interpret complex biological systems across molecular layers. As outlined in Table 1, integration methods range from classical statistical to deep generative learning approaches, each offering unique trade-offs in terms of interpretability, scalability, computational complexity, and generative capacity. The choice of an appropriate integration strategy depends on multiple factors, including the characteristics of the datasets, and the specific research and biological questions being addressed.

Classical approaches, such as correlation-based and matrix factorisation techniques, offer interpretable and computationally efficient solutions, making them well-suited for joint dimensionality reduction exploratory analyses or as foundational steps in more complex frameworks. Probabilistic models offer the benefit of explicitly modelling uncertainty, while kernel- and network-based methods enable integration in a unified sample similarity space, either through kernel functions or graph-based representations. Deep generative models, particularly VAEs, have shown considerable success in addressing the challenges of high-dimensional, sparse, and noisy data. These models are especially powerful for learning joint embeddings, imputation, and denoising. However, current implementations are largely focused on scRNA-seq and scATAC-seq data, leveraging their availability and complementarity, and the maturity of single-cell analysis workflows. While effective in these domains, this focus limits their generalisability to broader experimental settings. Furthermore, VAEs typically require greater computational resources and are sensitive to training data size. As model complexity increases through deeper networks, larger inner layers, or more sophisticated loss functions, GPU support and large memory are often needed. In the multi-omics context, where large datasets across omics layers and conditions are often scarce, model performance may degrade. Data scarcity can lead to unstable training, poor convergence, and reduced robustness, ultimately constraining the applicability of deep generative models in real-world biomedical scenarios.

While several benchmarking studies have examined the runtime and computational resource requirements of specific multi-omics integration tools [18, 41, 166–169], providing a precise comparative evaluation across all categories and methods remains challenging. Nevertheless, all approaches reviewed here are feasible to run on standard research computing infrastructures. As the field advances, continued efforts to enhance model generalisability, computational efficiency, and flexibility across diverse omics types will be essential. In this context, emerging paradigms such as foundation models and the integration of increasingly diverse data modalities hold great promise for enhancing the scope and impact of multi-omics research in precision medicine.

### Beyond omics: multimodal integration

**Figure 4:**
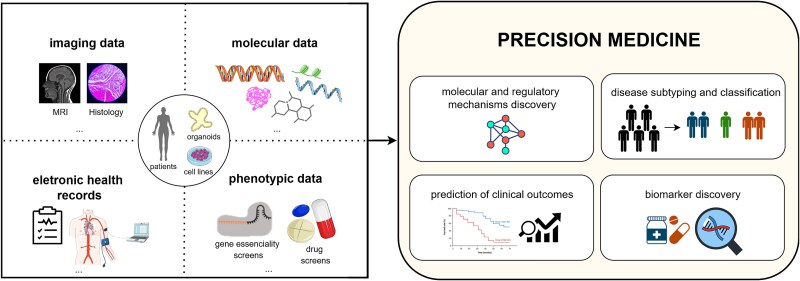
Integration of multiple data modalities—molecular, phenotypic, imaging and electronic health records datasets (left)—to uncover molecular and regulatory mechanisms, enable disease subtyping and classification, enhance clinical outcome predictions for diagnosis and prognosis, and identify biomarkers of therapeutic response (right).

The rapid advancement of biomedical technologies and the increasing diversity of data modalities present unprecedented opportunities for precision medicine and synthetic biology. Beyond molecular omics data, modern approaches increasingly integrate phenotypic datasets, imaging modalities, electronic health records, or bio-signals from wearable devices (Fig. 4). These multimodal datasets hold the promise of providing an even more holistic view of biological processes and diseases [170–172]. However, their integration remains challenging due to significant differences in data scale, format, and structure.

Integrating structured, tabular data, such as gene expression or clinical measurements, is comparatively straightforward, as these data modalities typically share compatible data distributions and can be aligned across samples. In contrast, unstructured data — such as spatially encoded medical images or clinical text, like electronic health records that require language models [173]—demands more advanced modelling strategies to extract meaningful biological insights.

Machine learning models have proven effective in this context. For example, Cheerla and Gevaert [174] developed a deep learning framework that integrates clinical data, molecular omics, and histopathology images using modality-specific FFNNs and a convolutional neural network (CNN) to learn a unified embedding used to predict survival through a Cox loss. Similarly, Carrillo-Perez et al. [175] proposed a late fusion strategy for non-small cell lung cancer (NSCLC) classification, where histology image features extracted by a CNN and molecular omics features modelled by support vector machines (SVMs) are later combined to produce the final output. Sammut et al. [176] introduced an ensemble machine learning model that integrates clinical, genomic, transcriptomic, digital pathology, and treatment data from breast cancer biopsies to predict therapeutic response. By combining modality-specific feature selection with an unweighted ensemble of logistic regression, SVMs, and random forests, the model achieved high predictive performance, validating the benefit of comprehensive multimodal data fusion.

In a more dynamic setting, DyAM [177], an attention-based multimodal framework designed to integrate clinical, genomic, radiomic features from CT scans, and pathology data for predicting immunotherapy response in NSCLC was proposed. DyAM processes each modality through logistic regression models and integrates them via a dynamic attention mechanism that adjusts weights based on modality relevance per patient. A masking function handles missing modalities by assigning them zero attention, enabling robust predictions without imputation.

Together, these studies highlight the increasing sophistication and potential of multimodal machine learning frameworks in precision medicine. Realizing this potential will require continued refinement of machine learning and advanced artificial intelligence (AI) methods to ensure their effective translation into clinical practice, ultimately enabling robust multimodal biomarker discovery and advancing data-driven, personalized patient care.

### Transformers and foundation models

Foundation models, initially designed for natural language processing [178], are now being expanded to biological datasets [179–183], demonstrating versatility in multi-batch and multi-omics integration, perturbation response prediction, and gene regulatory network inference. These models, typically based on the self-attention transformer architecture, are pre-trained on large, diverse datasets, enabling them to generalize across various domains. By leveraging transfer learning, the pre-trained model can be fine-tuned on a specific domain or task with much less data than what would be needed to train from scratch. Several transformers-based models for single-cell omics and foundation models for bioinformatics applications are reviewed in [184, 185].

In single-cell omics, scFoundation [182], pre-trained on tens of millions of scRNA-seq profiles, demonstrated strong performance across a variety of downstream tasks including cell type annotation, perturbation response prediction, and gene module inference. Similarly, scGPT [180], a generative pre-trained transformer trained on over 33 million single-cell transcriptomic profiles, supports batch correction, perturbation modelling, gene network inference, and is designed to extend toward multi-omics integration.

Recent advances increasingly emphasize explicit integration of multiple omics layers. For instance, mosGraphGPT [179] represents multi-omics profiles as molecular graphs and uses a generative transformer to predict disease phenotypes and uncover key signalling pathways. scmFormer [186] uses a multi-task transformer to integrate transcriptomics and proteomics, supporting label transfer, imputation, and spatial analysis. GET [183] is an interpretable foundation model for transcriptional regulation analysis that integrates chromatin accessibility and genomic sequence data to predict gene expression. It enables applications such as regulatory activity prediction and inference of transcription factor networks.

Beyond molecular profiles, foundation models have been extended to integrate biological networks [187, 188], pathway-level information [189], and unstructured clinical information such as electronic health records [173] or digital pathology data [190], broadening their impact in translational medicine.

A notable example is PathChat [191], a multimodal large language model (MLLM) designed for computational pathology. It integrates a vision encoder pre-trained on over 100 million histopathology image patches, with a 13-billion-parameter language model. To align image and pathology text representations, the encoder was further pre-trained on over 1.18 million pathology image–caption pairs. The combined MLLM was fine-tuned on over 450 000 pathology-specific instructions. PathChat is a vision-language AI assistant, capable of jointly interpreting pathology images and text, and achieves state-of-the-art performance on diagnostic tasks across diverse tissue types and disease contexts.

Despite their promise, concerns remain about the applicability and performance of foundation models compared to state-of-the-art machine learning approaches [192, 193], highlighting the need for further refinement of these models and careful identification of their most relevant applications. Nonetheless, foundation models represent a promising direction for integrating complex multimodal biological data and advancing multi-omics-driven discovery in precision medicine.

## Conclusions

This review explores diverse approaches to integrate multi-omics data, highlighting both classical methods and recent advancements in deep learning. We categorize integration techniques by their underlying approaches, offering a comprehensive technical overview of the models developed and highlighting their strengths, limitations, and applications. Classical methods remain valuable for their interpretability, serving as the foundational basis for the development of more complex models. Deep generative models, particularly VAEs, have significantly advanced the integration of high-dimensional and incomplete data, offering flexible designs and generative capabilities. Specifically, we review various regularisation strategies incorporated into VAE frameworks to address key challenges in multi-omics analysis and allow the extraction of biologically meaningful representations. Additionally, we highlight the emerging potential of foundation models and multimodal data to advance precision medicine research. Overall, this review provides a detailed overview of the current state of multi-omics integration methods and outlines promising directions for future advancements in understanding complex biological systems.

**Key Points**

- This review systematically covers multi-omics integration, from classical statistics to deep learning, emphasising on technical descriptions and standardisation of notations.
- Key focus in the generative potential of multi-omics models in precision medicine and synthetic biology, alongside recent technical advancements improving their performance.
- Given the central role of VAEs, we provide a detailed technical analysis of their architectures, loss functions, and regularisation, unifying terminology. This is the first systematic review of VAEs in multi-omics integration.
- We highlight emerging data modalities and foundation models, outlining future directions in multi-omics research.

## Data Availability

No new data were generated or analyzed in this review.
